# Synergistic benefits of AMF: development of sustainable plant defense system

**DOI:** 10.3389/fmicb.2025.1551956

**Published:** 2025-07-21

**Authors:** Gökhan Boyno, Younes Rezaee Danesh, Rojbin Çevik, Necmettin Teniz, Semra Demir, Emre Demirer Durak, Beatrice Farda, Amedeo Mignini, Rihab Djebaili, Marika Pellegrini, Rosa Porcel, José M. Mulet

**Affiliations:** ^1^Department of Plant Protection, Faculty of Agriculture, Van Yuzuncu Yil University, Van, Türkiye; ^2^Department of Agricultural Biotechnology, Faculty of Agriculture, Van Yuzuncu Yil University, Van, Türkiye; ^3^Department of Life, Health and Environmental Sciences, University of L'Aquila, L'Aquila, Italy; ^4^Instituto de Biología Molecular y Celular de Plantas, Universitat Politècnica de València-Consejo Superior de Investigaciones Científicas, Valencia, Spain

**Keywords:** microbial-based tools, beneficial fungi, arbuscular mycorrhizae, biocontrol, sustainable agriculture

## Abstract

Arbuscular mycorrhizal fungi (AMF) are a ubiquitous group of soil microorganisms that form symbiotic relationships with the roots of over 80% of terrestrial plant species. These beneficial fungi are crucial in plant growth, nutrition enhancement, and abiotic and biotic stress resilience. This review explores the AMF synergistic benefits including their capacity to interact with plant roots system to enhance nutrient absorption, improve stress resilience, and confer disease resistance, and their potential applications in sustainable agriculture. The Review integrates recent insights illustrating the molecular processes responsible for improving plant defense mechanisms by AMF, including the modulation of signaling pathways. It highlights the importance of AMF-induced systemic resistance in enhanced abiotic and biotic stress resistance. Moreover, the article provides an integrative perspective on applying AMF toward sustainable plant protection. Within this context, we discussed how these fungi improve plant performance, including enhanced nutrient acquisition, increased tolerance to environmental stressors, and enhanced protection against pathogens by improving plant resistance to biotic stress through the activation of the plant immune system. We also examine the ecological significance of AMF in maintaining soil health and fertility and highlight the importance of incorporating their management into sustainable agricultural practices. Future research directions and innovative applications are also presented. The literature survey demonstrated these fungi's versatility in improving plant tolerance to several biotic and abiotic stresses. At the scientific level, these abilities are supported by several open-field experiments on different plant species. Available commercial formulations and positive ongoing research of AMF, in combination with other sustainable tools, highlight the solid research outline on these beneficial fungi.

## 1 Introduction

The global population growth and climate change have increased the demand for sustainable agricultural practices to enhance crop productivity and mitigate the negative environmental impact (Rebello et al., 2021). Intensive agriculture has led to significant soil degradation and contamination, posing a major challenge (Gonzalez-Gonzalez and de-Bashan, 2023). Climate change has exacerbated the effects of abiotic stresses on crop productivity and ecosystem health, such as heat, salinity, drought, and heavy metal pollution (Begum et al., 2019). Additionally, environmental stresses heighten vulnerability to pests and diseases, undermining plant defence mechanisms and intensifying the occurrence and severity of these diseases. To address these issues, the potential to harness the power of rhizosphere microbiomes to develop innovative strategies for sustainable agriculture has been explored (Omomowo and Babalola, 2019). Plant-associated microbes play key roles in plant physiology, nutrient acquisition, and defense against biotic stressors. Several beneficial microorganisms have been found to provide ecological advantages while maintaining high production levels (Adeleke et al., 2024). However, high-input agriculture has been shown to alter the composition and diversity of these microbial communities (Kepler et al., 2020). Microbiome engineering refers to a targeted manipulation of microbes to enhance plant health and productivity. This customized approach involves the strategic addition of plant-derived compounds or direct application of microbial consortia designed to enhance crop growth and resilience (Arif et al., 2020). Recent studies have highlighted the potential of microbiome engineering to promote beneficial plant-microbe interactions and enhance crop yield and resilience (Cheng et al., 2023) Nevertheless, the success of microbial-based inoculants—a microbially produced formulations designed to introduce beneficial microbes into soil or plant environment—is contingent on understanding and manipulating the complex interactions between soil biotic and abiotic factors that shape the structure and function of these microbial communities (Bano et al., 2021). This approach can improve nutrient use efficiency, suppress plant pathogens, and mitigate abiotic stresses by harnessing the synergistic effects of plant-microbiome interactions. Emerging research has demonstrated the potential of rhizosphere microbiome engineering to revolutionize crop cultivation practices (Adeleke et al., 2024).

Arbuscular mycorrhizae fungi (AMF) represent a ubiquitous and ancient symbiotic relationship between soil fungi and plant roots, found in several terrestrial plant species (Rillig et al., 2016). These fungi can improve plant nutrient acquisition, particularly for phosphorus and other immobile nutrients, and enhance plant tolerance to biotic and abiotic stresses (Kepler et al., 2020). AMF colonization can also modify the composition and activity of the rhizosphere microbiome, leading to the recruitment of other beneficial microorganisms (Cheng et al., 2023). This symbiosis is necessary for nutrient uptake enhancement, improved stress tolerance, and the modulation of rhizosphere microbial communities (Bucking et al., 2012). AMF showed the ability to enhance plant growth, improve mineral nutrition, and increase tolerance to various abiotic and biotic stresses and plant disease (Lenoir et al., 2016). One of the key mechanisms by which AMF can confer enhanced resistance to pathogens is the induction of plant defense responses (Abdel-Fattah et al., 2011). The defense plant's recognition of specific signal molecules, or elicitors, during early stages of AMF colonization is an essential step for activating the plant's defense mechanisms. This can lead to the induction or suppression of various defense-related pathways, ultimately influencing the compatibility and development of the AMF-plant symbiosis (Garcia-Garrido, 2002). The increased in plant resistance to pathogens through AMF include: improvement of plant nutrient status, competition with pathogens for resources, changes in root morphology and structure, alterations in the rhizosphere microbial community, and the induction of local or systemic defense responses in the plant (Huang et al., 2003). AMF have also demonstrated a remarkable ability to enhance plant resilience against abiotic stresses, such as drought, salinity, and heavy metal contamination (Brar et al., 2024). This is achieved through various mechanisms, including improved water and nutrient uptake, production of stress-responsive compounds, and the modulation of plant physiology and gene expression (Delaeter et al., 2024). It has been reported that the inoculation of tomato plants with AMF *Funneliformis mosseae* significantly reduced early blight disease caused by *Alternaria solani* Sorauer (Song et al., 2015). Moreover, pre-inoculation of tomato with *Rhizophagus irregularis* enhanced plant resistance to *Fusarium oxysporum* through jasmonate (JA) biosynthesis pathway mechanism (Wang et al., 2022). Collectively, the synergistic benefits of AMF make these fungi a valuable asset for the development of sustainable agricultural practices that can address the challenges posed by climate change and environmental degradation (Chen et al., 2018). This review deals with the multifaceted functions of AMF in plant health and sustainable agriculture. It highlights how AMF triggers plant defense responses to abiotic and biotic stressors including pathogens, heavy metals, and drought. The synergistic interaction of AMF with various microbial agents and the additional ecological and environmental advantages of AMF applications, including increased soil health and reduced chemical inputs. Moreover, this review focuses on AMF's function in cropping production systems, highlighting their economy in cost-benefit ratio and sustainability. The review delves into the various strategies and challenges involved in harnessing the power of the AMF to develop sustainable and resilient agricultural systems.

## 2 The AMF plant defense responses triggering against biotic and biotic stressors

**Figure 1:**
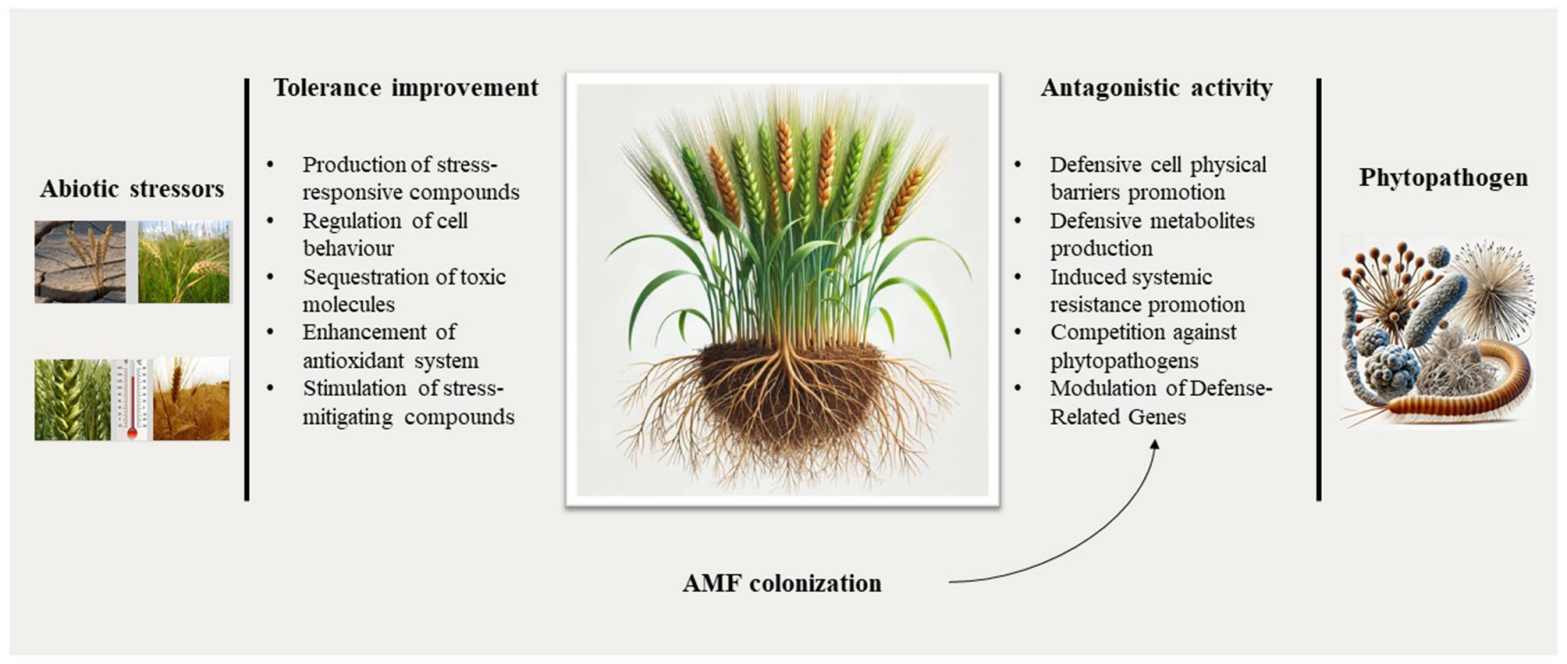
Summary of the plant defense mechanisms induced by AMF against abiotic and biotic stressors. Wheat plant is used as a representative example to illustrate these mechanisms. The icons of this image were created with the assistance of DALL·E.

One key mechanism by which AMF can enhance plant disease resistance is through the induction of defense responses. Figure 1 summarizes the plant defense mechanisms induced by AMF against biotic and biotic stressors. Upon colonization, AMF can trigger the accumulation of phenolic compounds and the activation of local or systemic defense pathways in the host plant. This activity can increase resistance against various plant pathogens, including soil-borne diseases (Abdel-Fattah et al., 2011). The ability of AMF to induce plant defense responses has been well-documented in various plant-pathogen systems. Studies have shown that AMF colonization can enhance the production of antimicrobial compounds, strengthen cell walls, and increase the activity of defense-related enzymes in the host plant (Weng et al., 2022). Moreover, AMF can also modulate the expression of defense-related genes, leading to the upregulation of key signaling pathways involved in plant immunity. In addition to direct defense mechanisms, AMF can also indirectly contribute to plant disease resistance by altering the composition and activity of the rhizosphere microbiome. AMF can suppress the growth of plant pathogens through competition for nutrients and space and by promoting the proliferation of beneficial microorganisms that can antagonize or outcompete the pathogens (Delaeter et al., 2024). These multifaceted effects of AMF on plant defense responses and the rhizosphere microbiome highlight their potential as a valuable tool for sustainable crop protection. AMF are considered an innovative solution for achieving sustainability in agricultural production (Schaefer et al., 2021). By establishing a symbiotic relationship with plant roots, AMF support plant growth and enhances the overall health of soil ecosystems. This symbiotic relationship facilitates plants' more efficient uptake of essential nutrients such as phosphorus, nitrogen, zinc, and copper. AMF play a crucial role in phosphorus mobilization, supporting plant development even in low nutrient conditions while also optimizing water uptake, thereby improving drought tolerance and resistance to abiotic stresses (Demirci et al., 2022; Wahab et al., 2023).

**Figure 2:**
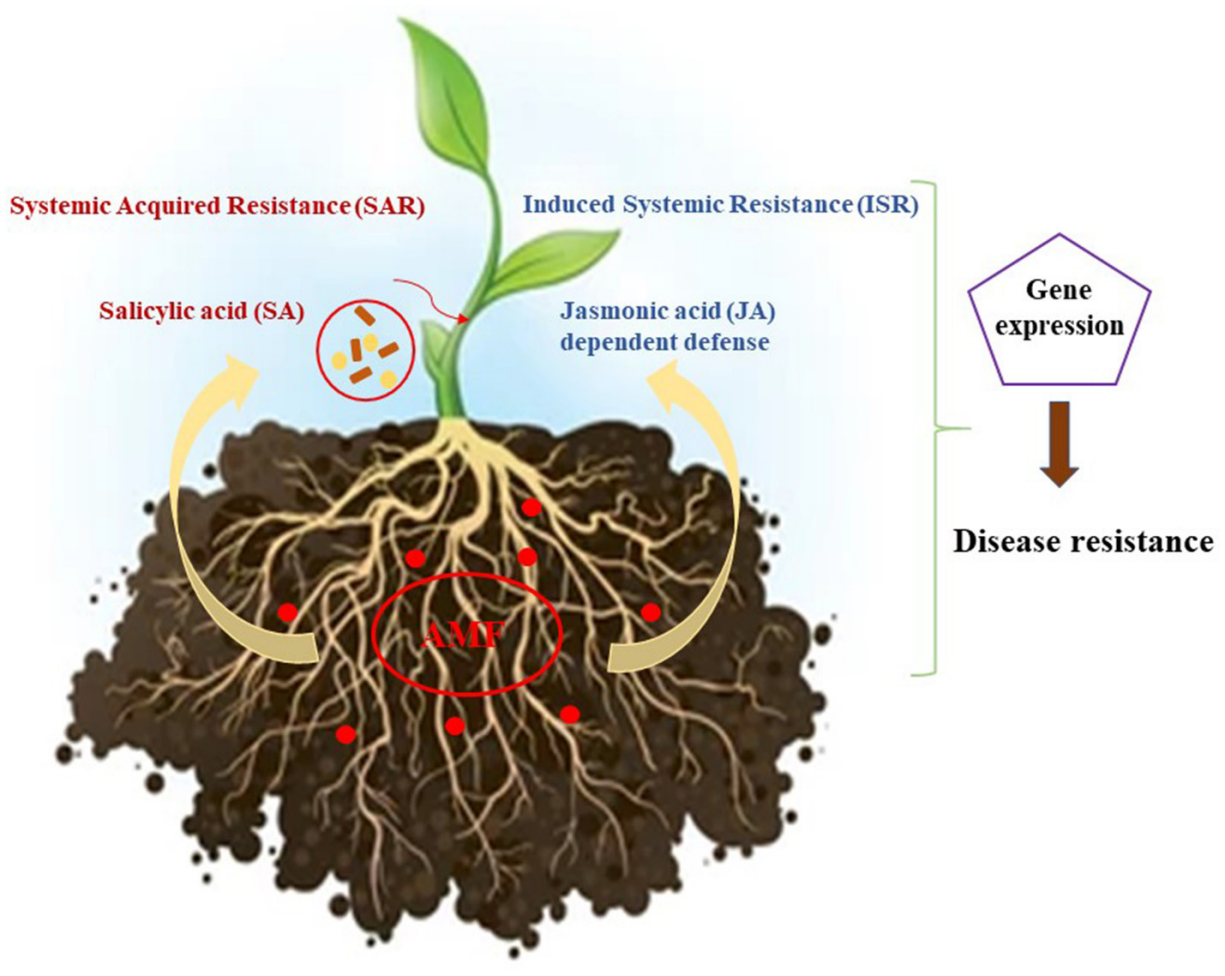
Graphical illustration on AMF influence on plant pathogen resistance through hormonal regulation pathways.

One of the most significant contributions of AMF is their ability to suppress plant diseases, functioning as a natural biological control agent. AMF protect against pathogens through two primary mechanisms (Figure 2). First, they form a physical barrier in the plant root system, making it more difficult for pathogens to penetrate plant tissues (Weng et al., 2022). Second, AMF stimulate plant defense mechanisms, contributing to the development of systemic resistance (Jung et al., 2012). By activating key defense signaling pathways, including salicylic acid (SA), jasmonic acid (JA), and ethylene, AMF enhance resistance against soil-borne pathogens (Weng et al., 2022). Moreover, AMF-induced thickening of plant root cell walls further restricts pathogen entry, effectively protecting against fungal and bacterial pathogens and helping to control fungal disease in strawberries (Demir et al., 2023).

AMF's positive impacts on soil health further underscore their importance in sustainable agriculture. The AMF glomalin protein enhances soil aggregate stability, preventing erosion and improving water retention capacity (Zhang et al., 2023). Additionally, AMF increase the release of carbon-based compounds from plant roots, which promotes microbial diversity in the soil, boosting its fertility and the long-term sustainability of ecosystems (Wen et al., 2024).

AMF applications also promote environmental sustainability by reducing the reliance on chemical fertilizers and pesticides. By mitigating soil and water pollution caused by pesticides and lowering the carbon footprint of agricultural practices, AMF help create more eco-friendly farming systems (Phour and Sindhu, 2024). Their ability to reduce dependency on chemical fertilizers offers both economic and environmental benefits (Lyu et al., 2021). These features highlight the potential of AMF to serve as biofertilizers and biocontrol agents in modern agricultural systems. However, one of the main challenges in AMF applications is managing the variations associated with different plant species and soil conditions (Fester and Sawers, 2011). Ensuring compatibility between AMF species and specific plant hosts and optimizing this symbiotic relationship requires further research. Additionally, the commercialization of AMF inoculants and the development of practical techniques for field applications are critical for their effective integration into agricultural practices (Boyno et al., 2024).

Beyond their role in enhancing plant resistance to biotic stress, AMF have also demonstrated remarkable abilities in improving plant tolerance to a wide range of abiotic stresses, including drought, salinity, and heavy metal contamination (Brar et al., 2024). AMF can help plants cope with these stresses through various mechanisms, such as improved water and nutrient uptake, the production of stress-responsive compounds, and the modulation of plant physiology and gene expression (Ruiz-Lozano et al., 2008; Delaeter et al., 2024). For instance, AMF can enhance plant drought tolerance by improving water and nutrient absorption, promoting the accumulation of osmoprotectants, and regulating stomatal behavior (Fontana et al., 2009). Similarly, AMF can alleviate the negative impacts of salinity and heavy metal stress by sequestering toxic ions, enhancing the antioxidant system, and stimulating the production of stress-mitigating compounds (Abd_Allah et al., 2017). The synergistic benefits of AMF, encompassing both biotic and abiotic stress tolerance, make these fungi a valuable asset for the development of sustainable agricultural practices. By harnessing the multifaceted capabilities of AMF, we can unlock new strategies for enhancing crop resilience and productivity in the face of the diverse challenges posed by a changing climate and environmental degradation (Faria et al., 2022). Extensive research has been conducted on the potential of AMF to improve plant performance and resilience, and the findings highlight the promising role of these fungi in sustainable agriculture. Table 1 summarizes the recent studies on open-field AMF application against biotic and biotic stresses.

**Table 1:** Recent studies concerning AMF open-field application against biotic and abiotic stressors.

**Host plant**	**AMF**	**Stress**	**Mechanism**	**References**
*Zea mays*	*Funneliformis mosseae, Rhizophagus irregularis, Rhizoglomus clarum*, and *Septoglomus deserticola*	*Diabrotica virgifera*	Disease severity decrease and crop yield improvement	Jaffuel et al., 2019
*Capsicum annuum*	*Rhizophagus aggregatus, Rhizophagus intraradices, Claroideoglomus etunicatum, Endogone mosseae, Funneliformis caledonium*, and *Gigaspora margarita*	Drought	Stress tolerance induction and growth and yield improvement.	Nurzyńska-Wierdak et al., 2021
*Manihot esculenta*	*Funneliformis mosseae* and *Glomus clarum*	Drought	Stress tolerance induction and plant development improvement	Oyetunji et al., 2007
*Coriandrum sativum*	*Glomus hoi*	Drought	Stress tolerance induction and plant development and essential oil improvement	Sani and Farahani, 2010
*Thymus vulgaris*	*Funneliformis mosseae*	Drought	Essential oil quality and quantity improvement	Amani Machiani et al., 2021
*Spinacea oleracea*	*Glomus* spp.	Drought	Plant growth and development improvement	Alotaibi et al., 2023
*Salvia officinalis*	*Funneliformis mosseae*	Drought	Plant development, essential oils and productivity improvement	Ostadi et al., 2022
*Triticum aestivum*	*Glomus etunicatum*	Drought	Plant growth and productivity improvement	Al-Karaki et al., 2004
*Elaeis guineensis*	*Glomus intraradices UT 126* and *Glomus clarum BR152B*	*Ganoderma boninense*	Disease severity decrease	Sundram et al., 2015
*Elaeis guineensis*	*Glomus deserticola*	*Ganoderma boninense*	Disease severity decrease and plant development improvement	Hendarjanti and Sukorini, 2022
*Metrosideros laurifolia*	*Claroideoglomus etunicatum* PSB1 and *Acaulospora rugosa* PS01 and RARC	Heavy metals (ultramafic soil)	Stress tolerance induction and plant growth improvement	Amir et al., 2019
*Arachis hypogaea*	*Rhizophagus irregularis* SA and *Funneliformis mosseae* BEG95	Salinity	Stress tolerance induction and yield and quality improvement	Qin et al., 2021
*Sorghum bicolor*	*Funneliformis mosseae*	*Striga hermonthica*	Infesting decrease and plant growth improvement	Isah et al., 2013
*Saccharum officinarum*	*Glomus etunicatum and Scutellospora fulgida*	*Striga hermonthica*	Infesting decrease and plant growth improvement	Manjunatha et al., 2018
*Sorghum sudanese*	*Glomus* spp.	Surface ozone	Stress tolerance induction and plant biomass improvement	Cui et al., 2013

As shown in Table 1, extensive applications of AMF in mitigating biotic and abiotic stresses across various plant species under field experiments were carried out. For instance, maize (*Zea mays*) benefits from AMF species such as *Funneliformis mosseae* and *Rhizophagus irregularis*, which reduce the severity of damage caused by *Diabrotica virgifera* and significantly improve crop yields (Jaffuel et al., 2019). Similarly, cassava (*Manihot esculenta*) exhibits enhanced drought tolerance and development when associated with *Funneliformis mosseae* and *Glomus clarum* (Oyetunji et al., 2007). Aromatic plants like thyme (*Thymus vulgaris*) and coriander (*Coriandrum sativum*) show improved essential oil production and quality under drought conditions with the aid of AMF such as *Funneliformis mosseae* and *Glomus hoi* (Sani and Farahani, 2010). Other examples include peanut (*Arachis hypogaea*), which achieves better yield and quality under salinity stress due to the association with *Rhizophagus irregularis* and *Funneliformis mosseae* (Qin et al., 2021), and wheat (*Triticum aestivum*), which benefits from *Glomus etunicatum* to improve growth and productivity during drought stress (Al-Karaki et al., 2004). Moreover, sorghum (*Sorghum bicolor*) sees decreased parasitic infestations caused by *Striga hermonthica* with AMF such as *Funneliformis mosseae* (Isah et al., 2013). These findings collectively underscore the ecological significance of AMF in enhancing plant health and productivity, making them indispensable for combating agricultural challenges in diverse environments.

## 3 Combined effects of AMF and other microbial communities in plant health

Soil microorganisms, especially in the rhizosphere, strengthen the resilience of plants against environmental pressures and offer a sustainable solution (Demir et al., 2022). Among them, Plant Growth Promoting Rhizobacteria (PGPR) are known to support plant root systems and improve nutrient acquisition efficiency through various biological mechanisms. Under stress conditions like drought, these microorganisms increase the water use efficiency of plants, reducing their dependence on water (Ullah et al., 2019). AMF is critical in increasing the bioavailability of essential nutrients like phosphorus. Although phosphorus is a crucial macronutrient for plants, its low bioavailability in soil often limits its uptake. AMF improves phosphorus availability, making it more accessible to plants (Bhantana et al., 2021). Additionally, AMF networks in plant roots facilitate the movement of water and nutrients in the soil, enabling more efficient nutrient acquisition (Kuyper et al., 2021). Rhizobacteria further enhance nutrient bioavailability, interact with plants to optimize nutrient uptake, and promote plant growth (Pii et al., 2015). Although the traditional vision is that phosphate and nitrogen are the most limiting macronutrients in agriculture, and that potassium is usually in excess, recent meta-analyses and studies, challenge this assumption (Brownlie et al., 2024; Fang et al., 2024). AMF can enhance the plant potassium uptake, not only for the well-established mechanisms in which the fungal hyphae can extend the plant's root system, allowing it to explore a larger volume of soil and access potassium sources that may be beyond the reach of the plant's roots (Shin, 2014). It has been shown that potassium and AMF can alter root morphology and nutrient uptake (Yuan et al., 2023). Recently it has been shown that the effect can be direct, AMF can activate root potassium channels such as *LbHAK* transporter gene in *Lycium barbarum* (Zhang et al., 2024). It is known that potassium uptake is a mechanism to modulate plant tolerance to abiotic stress (Zhang et al., 2024), and also, upon salt stress, AMF can alter the expression of Na and chloride channels (Wang et al., 2023), so this will further explain how AMF prevents abiotic stress in plants. The use of microbial inoculants containing PGPR, AMF, *Trichoderma* species or their combinations is becoming widespread (Vestberg and Cassells, 2009; Pathak et al., 2017). In addition to enhancing plant growth, these microbial communities enable plants to gain resistance to diseases and reduce the adverse effects of stress (Kumar and Verma, 2018). The interactions between AMF and rhizosphere bacteria form a complex and multifaceted synergy that significantly benefits plants (Wahab et al., 2023). This interaction includes mechanisms that protect plants against pathogens (Pérez-de-Luque et al., 2017). Rhizobacteria interact with plants to produce protective compounds, such as antimicrobial peptides, lactic acid, and siderophores, which limit the impact of pathogens in the soil or rhizosphere, thereby increasing plant resistance to diseases (Chepsergon and Moleleki, 2023). Certain rhizobacteria species also activate systemic defense pathways in plants, such as the salicylic acid signaling pathway, strengthening overall plant immunity (Saleem et al., 2021; Yu et al., 2022).

AMF complement these protective mechanisms by shielding roots from external threats. Mycorrhizal networks support root defenses and help isolate plants from pathogenic organisms, enhancing their ability to combat diseases (Boyno and Demir, 2022). Moreover, the interaction between rhizobacteria and AMF is linked to the production of phytohormones that directly promote plant growth (Pérez-de-Luque et al., 2017). Rhizobacteria can produce growth-regulating hormones, such as auxins, cytokinins, gibberellins, and ethylene. These hormones stimulate plant development, enhancing root and shoot growth and overall plant health (Tsukanova et al., 2017). AMF and rhizobacteria interactions can increase the production and effectiveness of these hormones, allowing plants to grow faster and healthier (Sbrana et al., 2014). Furthermore, AMF mycelial networks in roots facilitate the more effective distribution of phytohormones in the soil, improving plant growth responses (Boyno et al., 2023).

The mycorrhizal networks formed by AMF also enhance soil structure by binding soil particles and promoting the formation of aggregates, which improve soil aeration (Zeng et al., 2023). These networks also aid in water retention, minimizing plant water loss during drought. Rhizobacteria stabilize soil aggregates, further reinforcing soil structure and increasing its integrity (Naseem et al., 2018). These interactions significantly improve plant survival and growth, particularly under drought and other abiotic stress conditions (Dimkpa et al., 2009; Boyno and Demir, 2022). gPGPR also promotes the germination of AMF spores and the development of mycelia, which significantly impacts plant growth (Gopal et al., 2012). PGPR facilitates the efficient colonization of AMF in soil and rhizospheres, particularly under adverse conditions such as drought (Hnini et al., 2024). Prior research indicated that inoculation with PGPRs could enhance flavonoid accumulation in basil and lettuce plants (Jung and Kim, 2020; Dasgan et al., 2022). Flavonoids function as signaling molecules in mycorrhizal formation and demonstrate substantial positive correlations with AMF colonization of plant roots. These correlations likely indicate a crucial mechanism through PGP enhance AMF colonization rates in root systems (Zeng et al., 2025).

This facilitation enables AMF to establish themselves more quickly and effectively in plant roots, supporting plant growth under challenging conditions. However, some uncertainties and unresolved questions remain regarding the combined use of AMF and PGPR. While co-inoculated plants often show enhanced AMF colonization and improved plant growth, some studies have reported no significant increase in AMF colonization (Begum et al., 2022). These discrepancies may depend on plant and microorganism species, environmental conditions, and the specific effectiveness of the microorganisms under different circumstances. The combined use of plant growth-promoting rhizobacteria (PGPR) and AMF offers excellent potential, particularly in enhancing resistance to drought stress. It has been reported that the combined effect of AMF and PGPR improves tobacco plant growth and photosynthetic performance under drought stress through antioxidant defense mechanism and mineral nutrient metabolism enhancement (Begum et al., 2022). Moreover, a study conducted by Selvakumar and his collaborators showed that the interaction between AMF and spore associated bacteria (SAB) improved AMF symbiosis and alleviate salinity stress in Maize plant by regulating gene expression linked to ions transport and maintaining K^+^/Na^+^ homeostasis (Selvakumar et al., 2018).

However, the effects of AMF and PGPR interactions on pathogen resistance are not fully understood (Savastano and Bais, 2024). While some studies suggest that PGPR activate systemic defense pathways in plants to provide stronger protection against pathogens, this effect is not always correlated with increased AMF colonization (Savastano and Bais, 2024).

## 4 Effects of AMF on soil microbial diversity and function

AMF colonize plant roots, penetrate plant cell walls, and move into the cell membrane, establishing a mutually beneficial relationship with plants (Wahab et al., 2023). This symbiotic relationship supports plant growth and development by enhancing plant nutritional status and disease resistance. Rhizobacteria increase plant production by a variety of methods, including the release of plant hormones and secondary metabolic products, nutrient conversion into soluble forms, nitrogen fixation, and tolerance to both biotic and abiotic stressors. The symbiotic association between AMF and plants enhances plant growth and nutrient uptake across various soil and environmental conditions (Mazumder et al., 2025). This symbiosis elicits systemic resistance in plants, enhancing their tolerance to pathogen infections and mitigating the severity of plant illnesses (Ghorui et al., 2024). AMF can also improve the diversity and richness of rhizosphere microorganisms by enabling them to colonize the mycelium. The composition and activity of soil bacterial communities are often influenced by soil chemical characteristics (Huang et al., 2019). Nonetheless, they are significantly governed by rhizosphere root exudates or hyphal exudates within the hyphal ring. Consequently, soil microorganisms may be influenced by plant roots and arbuscular mycorrhizal fungi in various geographical niches (Wang et al., 2024).

AMF, as an endomycorrhizal fungus species, obtains organic carbon from plants to sustain its growth (Parihar et al., 2020). This relationship is essential for the survival of both the plants and the AMF.

AMF hyphae improve the plant's access to water and nutrients, particularly by increasing the bioavailability of elements such as phosphorus (Bhupenchandra et al., 2024). This process enables more efficient plant growth and development. Furthermore, the production of glomalin by AMF supports the formation of soil aggregates, improving soil structure and enhancing its water retention capacity (Rillig, 2004). AMF synthesizes glomalin, a glycosylated protein that serves as a structural element of hyphae and spore walls (Singh et al., 2016). Glomalin, secreted by AMF's exterior hyphae and spore walls, facilitates the adhesion of soil particles to other organic matter (Singh et al., 2013). Glomalin accumulates in the soil matrix following fungal senescence and turnover, and it is regarded as a pervasive component of soil organic matter linked to a broad distribution of AMF (Magurno et al., 2019). Glomalin plays a vital role in various ecosystem functions, including soil aggregation, carbon storage, nutrient cycling, soil biodiversity, stabilization of heavy metals and organic contaminants, and ecological restoration. Glomalin serves as a direct source of nutrients for microorganisms and plants. Glomalin has been shown to promote soil aggregate stability by increasing the bond energy of aggregates, especially macroaggregates (Ji et al., 2019). Furthermore, an elevation in glomalin concentration in soil enhanced many physical attributes, including reduced bulk density and increased soil porosity, moisture content, and water-holding capacity. Glomalin also increases nutrient bioavailability in the soil, strengthens microbial efficiency, and boosts soil fertility (Zhang et al., 2023).

The interaction between AMF and plants enhances nutrient and water uptake and strengthens plant defense mechanisms. While forming a symbiotic relationship with plants, AMF regulates defense responses against pathogens (Garcia-Garrido, 2002). Mycorrhizal colonization activates the plant's defense genes, providing stronger resistance, particularly against fungal and bacterial pathogens (Jung et al., 2012). AMF favors the proliferation of beneficial bacteria over pathogens in the rhizosphere through Shifting root exudates to promote beneficial microorganisms, production of secondary metabolites acting against pathogens, induction of plant immune system, competition with pathogens for space and nutrients, and enhancing plant growth and resilience (Rodríguez-Caballero et al., 2017). The AMF promotes water and nutrient assimilation via a filamentous hyphal network, resulting in enhanced soil structure (Varinderpal-Singh et al., 2020). Rhizobacteria retrieve inaccessible nutrients from the rhizosphere, fix atmospheric nitrogen, or promote plant growth through hormone synthesis, suppression of soil-borne diseases, or enhancement of stress resistance in plants (Varinderpal-Singh et al., 2020). The function of PGPR in solubilising inaccessible forms of phosphorus in soil through acidification, chelation, exchange reactions, and the secretion of organic acids and phosphatases is well established (Maldonado et al., 2020). When combined, the AMF and PGPR can enhance nutrient availability and have positive impacts on the physical and biological characteristics of soil (Varinderpal-Singh et al., 2020). This interaction helps plants become more resistant to diseases. The interplay between arbuscular mycorrhizal fungi and plants frequently entails intricate signalling pathways that intersect with those associated with induced systemic resistance. The interaction between the signalling pathways of AMF colonisation and ISR induction can collaboratively enhance plant defense responses (Badrbani et al., 2024; Hussain et al., 2024). The formation of priming is one method by which arbuscular mycorrhizal fungi (AMF) might influence the efficacy of the induced systemic resistance (ISR) in plants. When plants form a symbiotic association with arbuscular mycorrhizal fungi, they experience alterations in gene expression and metabolic pathways that can induce the activation of defense-related genes. The priming effect enables the plant to respond more rapidly and efficiently when confronted by diseases (Kadam et al., 2020; Badrbani et al., 2024). Additionally, it has been shown that AMF can regulate biochemical defense pathways, triggering plants to produce more protective compounds (Zou et al., 2021). The impact of AMF and Rhizobacteria inoculants on plant yield, carbon sequestration, nutrient dynamics, microbial diversity, and potential microbial community functions have been documented (Meng et al., 2015; Püschel et al., 2017; Calderon and Dangi, 2024).

AMF has a broad impact on soil microbial communities. When these microorganisms interact with plant roots, they change the diversity and population structure of microorganisms in the rhizosphere. Mycorrhizal colonization alters the composition of soil microbes while providing additional nutrient sources to other microorganisms, mainly bacteria, and other fungi (Miransari, 2011). This interaction accelerates nutrient cycling in the soil, facilitates organic matter decomposition, and strengthens ecosystem functions. Moreover, AMF has been shown to enhance soil biodiversity by altering the structure of microbial communities through microbial inoculation and increasing the size of mycorrhizal colonies (Powell and Rillig, 2018).

AMF's propagation depends on the presence of soil microorganisms and the nature of their relationships with these organisms. Advanced techniques, such as stable isotope analysis, have revealed that AMF can coordinate nutrient exchange between microorganisms and mycorrhizae after forming a symbiotic relationship with plants (Duan et al., 2024). This mechanism improves nutrient cycling in soil ecosystems and supports soil health by enhancing the functions of microorganisms. Notably, AMF has been shown to increase organic matter accumulation in the soil and boost the biological activity of soil microorganisms (Zhou et al., 2020).

## 5 Multiple microbial agents in plant health: AMF and other microbial agents

Soil-borne plant pathogens, mainly fungi and bacteria, require long-term and effective management. Success in this management can be achieved through cultural, chemical, biological, and physical methods. However, due to the challenges of controlling soil-borne diseases with chemical pesticides in continuous cropping systems, the development of green technologies has become very important (Panth et al., 2020). The rhizosphere is a critical area where plant nutrient acquisition actively occurs and where plant-soil-microorganism interactions are intense (Bakker et al., 2012). These interactions, which affect the development of soil-borne diseases, are shaped by the colonization of plant roots by various symbiotic microorganisms, leading to strong competitive, and supportive interactions among them (Niu et al., 2020). Therefore, investigating the effects and mechanisms of different combinations of microbial agents is of significant theoretical and practical importance.

In the past, biological control agents were typically applied using a single microorganism species. However, recent studies have shown that using multiple microbial agents together leads to more potent and more effective results (Djebaili et al., 2021; Stenberg et al., 2021; Demir et al., 2023; Rezaee Danesh et al., 2024). Plant roots are colonized by symbiotic microorganisms such as mycorrhizal fungi, endophytic fungi (DSE), Trichoderma species, and plant growth-promoting rhizobacteria (PGPR). These microorganisms can prevent pathogen infections and promote plant growth. Some biological control agents effectively control pathogens, but when combined with other agents, they show a more significant impact. For example, a field study demonstrated that applications with arbuscular mycorrhizal fungi (AMF) and *Bacillus* sp. allowed a 50% reduction in the recommended NPK fertilization without compromising crop growth, nutrition, and yield (Nanjundappa et al., 2019). Combined inoculation with *Funneliformis mosseae* and Bacillus sp. M3-4 or *Glomus versiforme* and *Bacillus* sp. M3-4 promoted potato growth and triggered an enhanced defense response to control bacterial wilt disease (Shu-peng et al., 2015). In a greenhouse trial conducted by Liu and Zhang (Liu and Zhang, 2021), combinations of AMF *F. mosseae, Rhizophagus intraradices*, and *G. versiforme* with different PGPR strains were tested for their effects on plant growth, resistance to Fusarium wilt, and cucumber yield. The most effective combinations, Fm + PS1-5, Fm + PS3-2, and Gv + PS2-6, significantly controlled the *Fusarium* wilt.

Mutualisms between plant + fungus, plant + bacteria, and fungus + bacteria are mutualistic symbiotic systems that create a composite symbiont, even in natural ecosystems. These combined symbionts can perform more substantial functions than when applied alone. Field trials have shown that the AMF + *Trichoderma longibrachiatum* combination effectively reduces the incidence of soil-borne diseases and improves crop quality (Yang et al., 2022). Wang et al. (2019) observed in a field trial that the *T. harzianum* + *Bacillus cereus* combination provided better control over tomato root-knot nematodes. *T. harzianum* inhibits *Rhizoctonia solani* through parasitism, while *Bacillus subtilis* antagonistically inhibits the same pathogen. Combining these different inhibition mechanisms prevented *Alternaria solani*'s impact on potato black scurf disease (Boyno et al., 2022).

When symbiotic microbial agents are used together, they can regulate plant physiological metabolism and demonstrate their effects. Inoculating *Fragaria* × *ananassa* plants with mycorrhizal preparations such as Mykoflor (containing *Rhizophagus irregularis, F. mosseae*, and *Claroideoglomus etunicatum*), MYC 800 (containing *R. intraradices*), and the bacterial preparation Rhizocell C (containing *Bacillus amyloliquefaciens* IT45) increased the transpiration rate in intercellular spaces in leaves and elevated CO2 concentration (Mikiciuk et al., 2019). Additionally, these treatments increased the soil's total bacterial and fungal populations, enhancing plant efficiency. Plants treated with MYC 800 + Rhizocell C showed a higher CO2 assimilation rate than the control. The AMF + PGPR combination antagonizes pathogens, stimulates the synthesis of disease resistance signaling molecules, increases the expression of defense genes, boosts defense enzyme activities, and reduces the accumulation of toxic substances, thereby decreasing the incidence of cucumber *Fusarium wilt* disease and improving plant disease resistance (Yue et al., 2017).

## 6 Ecological and environmental benefits of AMF applications

AMF possess unique characteristics that provide multifaceted benefits to agriculture and ecosystem management. By establishing a symbiotic relationship with plant roots, AMF not only promote plant growth but also enhance plants' adaptation to stress conditions, thereby increasing environmental resilience (Begum et al., 2019). This symbiosis forms an extensive hyphal network that expands the root-soil interface, enabling more efficient uptake of essential macro- and micronutrients such as phosphorus and nitrogen (Mishra et al., 2024). Through increasing phosphorus solubility, AMF help plants to develop resistance against abiotic stresses including drought, salinity, and heavy metal contamination (Khan et al., 2023). These mechanisms are critical for maintaining productivity in agricultural systems challenged by both biotic and abiotic stressors (Berruti et al., 2016). Beyond promoting plant growth and stress tolerance, AMF significantly contribute to soil health and environmental sustainability. One of the key factors is glomalin, a protein produced by AMF that enhances soil aggregate stability, thereby strengthening soil structure and reducing erosion risks (Zhang et al., 2023). Glomalin also contributes to increasing soil organic matter content, improving water retention capacity, and enhancing soil carbon sequestration potential (Zhang et al., 2022). These properties position AMF a valuable tool in reducing the carbon footprint of agricultural practices and combate climate change (Rillig, 2004). The soil structure improvement also underpins the long-term preservation of agricultural productivity, especially in intensively farmed regions. In addition to these, soil-related benefits, AMF reduce reliance to chemical fertilizers and pesticides, promoting a more sustainable agriculture practice (Aggarwal et al., 2011; Srivastava et al., 2017). Through their symbiotic association with plant roots, AMF mobilize nutrients like phosphorus, which are often limited in soils, AMF decrease the need for phosphate fertilizers and other chemical inputs (Ibrahim et al., 2022). This natural nutrient acquisition not only lowers production costs but also mitigates soil and water pollution associated with excessive fertilizer use (Bhantana et al., 2021). Moreover, the biological barrier formed by AMF around plant roots acts as a natural defense against pathogens, reducing the necessity for pesticide applications (Harrier and Watson, 2004). Consequently, AMF contribute directly to minimizing environmental damage while facilitating the adoption of eco-friendly farming system (Gosling et al., 2006). Overall, AMF's ability to mitigate the use of chemicals serves as a model for sustainable practices, tying nutrient management to ecological conservation and leading the way toward more sustainable and ecologically resilient farming practices. Importantly, AMF also enhance ecosystem biodiversity through their interaction with the plant root microbiome. By promoting the establishment and proliferation of beneficial microorganisms, AMF improve plant health and maintain biological balance within soil ecosystems (Wahab et al., 2023; Hnini et al., 2024). The biological equilibrium fostered by AMF is therefore vital not only for agricultural landscapes but also for preserving ecosystem services in natural environments (Mwampashi et al., 2024). Overall, these multifaceted benefits demonstrate that AMF are essential agents for growth promotion, cost-reduction, and environmental conservation in agriculture. The broad applicability offers a strategic approach to enhancing agricultural productivity while strengthening the ecosystems sustainability (de Oliveira et al., 2024). Thus, the widespread adoption of AMF-based technologies is a key tool for achieving environmental and ecological sustainability in modern agriculture.

Despite the significant benefits of AMF in enhancing plant nutrient uptake and reducing the need for chemical fertilizers, the use of AMF is constrained by several factors. First, the symbiotic effectiveness of AMF is greatly influenced by soil properties, including pH, texture, nutrient content, and microbial community composition, and can vary significantly from site to site (Angelard et al., 2014; Igiehon and Babalola, 2017). Moreover, AMF are obligate symbionts that cannot be grown in pure cultures separate from their host plants. This limiting factor renders the large-scale manufacture of AMF inocula highly challenging and intricate. There are three primary categories of AMF inocula. Initially, soil from the root zone of a plant associated with AMF might serve as inoculum, as it often comprises colonized root fragments, AMF spores, and hyphae. Nevertheless, in the absence of accurate data regarding propagule abundance, diversity, and infectivity, soil inocula may prove unreliable, posing a potential risk of transmitting weed seeds and diseases. Spores obtained from soil can be utilized as initiators for the generation of crude inoculum. Crude inoculum can be acquired by cultivating a known isolate of AMF alongside a host trap plant, which is capable of extensive colonization by many AMF species, in an inert medium specifically optimized for AMF propagation (IJdo et al., 2011; Berruti et al., 2016). Additionally, AMF inoculants can range in price according to formulation and quality. This is the predominant type of inoculum used or large-scale crop inoculation, as it often comprises a more concentrated array of the same propagules seen in soil inocula. Infected root fragments from a recognized AMF host, isolated from a trap plant culture, can also function as a source of inoculum (Berruti et al., 2016).

Moreover, not all crop cultivars can establish equally effective symbiosis with AMF, and some of the more recent high-yielding cultivars can be less responsive as a result of selection under high-fertilizer application. Moreover, environmental conditions such as temperature, water, and seasonal variations influence AMF colonization and activity. Practical limitations also persist in the large-scale production of inoculum, formulation, storage, and field delivery, which affect the consistency and reliability of AMF-based biofertilizers. Hence, while AMF provide a valuable option for sustainable agriculture, their application requires careful adaptation to local agroecosystems to derive maximum benefits and avoid potential constraints (Igiehon and Babalola, 2017).

## 7 AMF and their roles in conventional and organic farming

Arbuscular mycorrhizal fungi (AMF) are recognized as an indispensable component of sustainable agricultural practices in both conventional and organic farming systems (Gosling et al., 2006). These microorganisms support plant growth and significantly contribute to environmental sustainability by reducing reliance on chemical inputs. In conventional agriculture, one of the primary functions of AMF is to solubilize and transport nutrients such as phosphorus, zinc, and other micronutrients that are otherwise difficult for plants to access. This capability significantly reduces the need for chemical fertilizers like phosphate fertilizers (Bindraban et al., 2020). Excessive phosphorus application can lead to environmental issues such as groundwater pollution and eutrophication. By mitigating these problems, AMF lower agricultural production costs while minimizing environmental risks (Jansa et al., 2006). Moreover, AMF serve as an effective tool for biological control of plant diseases in conventional farming. They protect plants from soil-borne pathogens, reducing infection rates from harmful organisms such as *Verticillium dahliae* and *Fusarium oxysporum* (Kowalska, 2021; Meddad-Hamza et al., 2023). This protection is achieved through two primary mechanisms. The first is forming a physical barrier in plant roots, which hinders pathogen entry (Weng et al., 2022). The second mechanism involves the activation of plant defense systems, aiding in the development of systemic resistance. AMF-induced defense mechanisms enhance the production of plant hormones such as salicylic acid and jasmonic acid, thereby bolstering resistance against pathogens (Liu and Chen, 2024). Such biological control strategies reduce the dependency on chemical pesticides, minimizing their adverse environmental impacts.

In organic farming systems, the importance of AMF is even more pronounced—organic farming limits chemical fertilizers and pesticides, aiming to maximize the benefits of natural resources. AMF play a critical role in these systems as part of natural fertilization processes (Berruti et al., 2016). By accelerating the decomposition of organic matter and supplying biologically available nutrients to plants, AMF enhance the productivity of organic farming systems. Practices like composting and green manure applications stimulate AMF colonization, enriching soil microbial diversity, supporting plant growth, and strengthening soil health and ecosystem balance (Gujre et al., 2021).

One of the most significant advantages of AMF in organic farming is their compatibility with eco-friendly practices and natural ecosystems (Phour and Sindhu, 2024). AMF play a key role in suppressing pathogens and improving plant health in organic systems. For instance, AMF integrate into plant root systems to enhance defense responses while promoting the establishment of beneficial microbial communities in the soil (Liu and Chen, 2024).

Additionally, AMF's ability to enhance carbon sequestration significantly reduces organic farming systems' carbon footprint (Avasiloaiei et al., 2023).

In both farming systems, the multifaceted benefits of AMF promote the adoption of sustainable approaches in agricultural production. In conventional farming, AMF reduce the use of chemical inputs, resulting in economic savings while supporting sustainability by minimizing environmental risks (Vosátka and Albrechtová, 2009). In organic farming, AMF optimize natural processes, improving plant growth and disease management, thereby enhancing these systems' efficiency and environmental compatibility (Gosling et al., 2006).

## 8 Application of AMF and their economic advantages

The application of arbuscular mycorrhizal fungi (AMF) in agriculture offers significant economic advantages both in the short and long term, enhancing cost efficiency in agricultural production systems. In the current era, where rising farming costs and the need for sustainable resource management are pressing challenges, biological solutions like AMF have become increasingly important (Fester and Sawers, 2011). One of the most notable contributions of AMF is their ability to reduce the use of chemical fertilizers and pesticides, thereby lowering production costs while promoting environmental sustainability (Igiehon and Babalola, 2017). By transporting nutrients such as phosphorus and zinc to plant roots, AMF decrease reliance on phosphate fertilizers. As phosphorus prices continue to rise and access is limited, the use of AMF stands out as a sustainable solution that alleviates the economic burden on farmers (Chen et al., 2018).

The economic benefits of AMF are not limited to reducing input costs; they also contribute to increased crop yield and quality, directly boosting farm income. AMF promote plant growth and enhance plant resilience under abiotic stress conditions (Demir et al., 2022). This enhancement leads to healthier, more robust, and higher-yielding crops. Improved tolerance to stress factors such as drought and salinity helps minimize crop losses caused by natural disasters, thereby reducing farmers' economic losses (Wahab et al., 2023). Increased yields directly enhance total revenue, while improvements in crop quality provide a valuable advantage, especially in premium markets. High-quality produce can command higher prices, increasing farmers' profitability and providing a competitive edge in the marketplace (Gosling et al., 2006).

One of the most critical long-term economic benefits of AMF is their role in improving soil health and supporting its sustainable use. Glomalin, a soil protein produced by AMF, enhances soil organic matter, strengthens soil aggregate stability, and prevents erosion (Channavar et al., 2024). Such improvements in soil structure preserve agricultural land's long-term productivity and reduce the restoration costs associated with land degradation. Healthy and fertile soils play a crucial role in ensuring the long-term economic sustainability of farming operations and increasing the value of agricultural land (Powlson et al., 2011).

The economic potential of AMF is further amplified by the growing consumer demand for environmentally friendly agricultural products. By reducing environmental impacts and supporting sustainable farming practices, AMF provide a competitive advantage for farmers adopting organic and eco-friendly farming methods (Phour and Sindhu, 2024). The increasing demand for organic products offers farmers opportunities for higher income while promoting sustainable agricultural practices (Hamel and Strullu, 2006). This trend translates environmental awareness into economic advantages for producers and consumers.

## 9 Conclusions, future research directions, and innovative applications

In addition to the widely reported benefits associated with AMF, the present review identifies the importance of AMF and plants in activating the defensive systems. AMF symbiosis has a direct influence on the expression of the most important defense-related genes in the plant, thereby enhancing the plant's response to abiotic and biotic stressors. The dynamic interaction activates a sustainable plant defense mechanism through systemic resistance and overall plant health. Accordingly, the molecular interaction between plant defense systems and AMF provides feasible opportunities for developing sustainable strategies to enhance farm protection. Mycorrhizal relationships strengthen critical ecosystem processes, such as nutrient cycling and organic matter decomposition, thereby improving soil health. Understanding these interactions is crucial in enhancing soil fertility and developing sustainable agricultural practices. AMF's potential to improve biodiversity and ecosystem functions within the soil is critical in supporting plant health and ensuring environmental sustainability. AMF provide a promising biotechnological solution for sustainable agriculture from both environmental and economic perspectives. By enhancing plant growth, protecting against pathogens, and improving soil health, AMF contribute to increased agricultural productivity while minimizing environmental impacts. Although the benefits of AMF are well-documented and commercial formulations are available, there are ongoing opportunities for further research and innovative applications to enhance their impact on sustainable agriculture. A possible future direction lies in the genetic improvement of AMF genomes to enhance their symbiotic efficiency, ecological adaptation, and functional superiority. The most recent advances in molecular biology and biotechnology, such as genome sequencing tools, gene editing tools (e.g., CRISPR/Cas9), and transcriptome profiling, offer unparalleled possibilities for analyzing the genetic basis of AMF-plant interactions. Investigators should focus on producing or screening AMF strains that perform better in additional abiotic conditions such as drought, salinity, and temperature by identifying dominant genes related to nutrient transfer, abiotic stress tolerance, and defense signaling. Genetic improvement can also make AMF more effective at colonizing a wide range of host plants and soils than current limitations in specificity and environmental compatibility. Genetic modification of stress-tolerant, high-yielding AMF strains using such molecular techniques would revolutionize their use in sustainable agriculture as tailored bioinoculants for agroecosystem. This synergy of cutting-edge genomics with traditional mycorrhizal research holds a potential for revealing new insights to enhance plant health, defense, and crop productivity (Shaw and Etterson, 2012). Another promising research direction is the exploration of novel delivery systems and application methods for AMF inocula. Current techniques, such as seed coatings or soil drenches, may not always provide optimal distribution and colonization of AMF within the rhizosphere. Innovative approaches (e.g., smart nanoparticle-based carriers or integrating AMF into biofilm-forming bacterial consortia) could enhance the targeted delivery and persistence of AMF in the soil. These improvements could lead to more efficient and consistent plant root colonization, improving crop growth, and health. Continuous research and innovation in this field will lead to even more significant advancements in sustainable agricultural farming practices.
